# Supplementary material for: Modeling glioblastoma heterogeneity as a dynamic network of cell states
Source: Mol Syst Biol. 2021 Sep 16;17(9):e10105. doi: 10.15252/msb.202010105 (PMC8444284; doi:10.15252/msb.202010105)
Supplement: Supplementary file 6 — Source Data for Figure 5 [file MSB-17-e10105-s004.zip › Figure5A_sourcedata/GSEA_3071/nosigdb_stateVI.GseaPreranked.1622795169056/RICHARDS_NOWAKOWSKI_SCIENCE_2017_MGE_IPC2_UPREG.html]

Details for gene set RICHARDS\_NOWAKOWSKI\_SCIENCE\_2017\_MGE\_IPC2\_UPREG[GSEA]

|  || Dataset | state63071 |
| Phenotype | NoPhenotypeAvailable |
| Upregulated in class | na\_neg |
| GeneSet | RICHARDS\_NOWAKOWSKI\_SCIENCE\_2017\_MGE\_IPC2\_UPREG |
| Enrichment Score (ES) | -0.5132841 |
| Normalized Enrichment Score (NES) | -2.8605757 |
| Nominal p-value | 0.0 |
| FDR q-value | 0.0 |
| FWER p-Value | 0.0 |
Table: GSEA Results Summary

  

Fig 1: Enrichment plot: RICHARDS\_NOWAKOWSKI\_SCIENCE\_2017\_MGE\_IPC2\_UPREG      
 Profile of the Running ES Score & Positions of GeneSet Members on the Rank Ordered List

  

| PROBE | GENE SYMBOL | GENE\_TITLE | RANK IN GENE LIST | RANK METRIC SCORE | RUNNING ES | CORE ENRICHMENT || 1 | CDC20 |  |  | 15 | 1.073 | 0.0019 | No |
| 2 | RND3 |  |  | 49 | 0.866 | -0.0028 | No |
| 3 | KCTD9 |  |  | 115 | 0.712 | -0.0179 | No |
| 4 | EIF3D |  |  | 152 | 0.663 | -0.0247 | No |
| 5 | SLC38A1 |  |  | 178 | 0.622 | -0.0284 | No |
| 6 | CCNB1 |  |  | 181 | 0.620 | -0.0253 | No |
| 7 | CLIC1 |  |  | 258 | 0.561 | -0.0446 | No |
| 8 | PTTG1 |  |  | 344 | 0.514 | -0.0669 | No |
| 9 | TROAP |  |  | 479 | 0.461 | -0.1041 | No |
| 10 | TUBB6 |  |  | 539 | 0.444 | -0.1191 | No |
| 11 | KITLG |  |  | 584 | 0.430 | -0.1296 | No |
| 12 | ARL4C |  |  | 666 | 0.408 | -0.1513 | No |
| 13 | RAP2B |  |  | 802 | 0.376 | -0.1893 | No |
| 14 | HERPUD1 |  |  | 830 | 0.371 | -0.1952 | No |
| 15 | CORO1C |  |  | 873 | 0.365 | -0.2055 | No |
| 16 | DDX39A |  |  | 936 | 0.355 | -0.2219 | No |
| 17 | KIF20A |  |  | 961 | 0.351 | -0.2269 | No |
| 18 | CCNB2 |  |  | 967 | 0.350 | -0.2263 | No |
| 19 | PRR11 |  |  | 969 | 0.350 | -0.2246 | No |
| 20 | COPS8 |  |  | 994 | 0.347 | -0.2296 | No |
| 21 | UPF3B |  |  | 998 | 0.346 | -0.2285 | No |
| 22 | CENPW |  |  | 1019 | 0.342 | -0.2324 | No |
| 23 | HIST1H2BD |  |  | 1045 | 0.338 | -0.2378 | No |
| 24 | CCND2 |  |  | 1064 | 0.335 | -0.2412 | No |
| 25 | TMEM126B |  |  | 1227 | 0.312 | -0.2876 | No |
| 26 | CDC27 |  |  | 1259 | 0.308 | -0.2950 | No |
| 27 | NEK2 |  |  | 1307 | 0.302 | -0.3072 | No |
| 28 | SAE1 |  |  | 1340 | 0.299 | -0.3150 | No |
| 29 | HDGF |  |  | 1502 | 0.279 | -0.3613 | No |
| 30 | DEPDC1 |  |  | 1545 | 0.275 | -0.3722 | No |
| 31 | BIRC5 |  |  | 1667 | 0.263 | -0.4067 | No |
| 32 | SEPT10 |  |  | 1724 | 0.258 | -0.4219 | No |
| 33 | IGF2BP1 |  |  | 1772 | 0.254 | -0.4344 | No |
| 34 | HAUS8 |  |  | 1808 | -0.250 | -0.4433 | No |
| 35 | ZNF738 |  |  | 1811 | -0.251 | -0.4424 | No |
| 36 | RBM23 |  |  | 1824 | -0.252 | -0.4445 | No |
| 37 | MKI67 |  |  | 1825 | -0.253 | -0.4430 | No |
| 38 | RTKN2 |  |  | 1850 | -0.256 | -0.4486 | No |
| 39 | HMGN2 |  |  | 1857 | -0.258 | -0.4489 | No |
| 40 | CKAP2L |  |  | 1884 | -0.262 | -0.4550 | No |
| 41 | STAG1 |  |  | 1895 | -0.263 | -0.4565 | No |
| 42 | GEN1 |  |  | 1899 | -0.263 | -0.4558 | No |
| 43 | ARL6IP6 |  |  | 1908 | -0.264 | -0.4566 | No |
| 44 | SMARCA5 |  |  | 1910 | -0.264 | -0.4553 | No |
| 45 | WDHD1 |  |  | 1923 | -0.267 | -0.4573 | No |
| 46 | MND1 |  |  | 1943 | -0.270 | -0.4614 | No |
| 47 | HIST1H3D |  |  | 1946 | -0.270 | -0.4604 | No |
| 48 | USP33 |  |  | 1947 | -0.270 | -0.4587 | No |
| 49 | PCDH17 |  |  | 1955 | -0.271 | -0.4592 | No |
| 50 | FANCD2 |  |  | 1956 | -0.271 | -0.4576 | No |
| 51 | NUP62 |  |  | 1960 | -0.272 | -0.4569 | No |
| 52 | AURKB |  |  | 1978 | -0.274 | -0.4603 | No |
| 53 | EFS |  |  | 1988 | -0.276 | -0.4613 | No |
| 54 | KIF22 |  |  | 2014 | -0.281 | -0.4671 | No |
| 55 | ACYP1 |  |  | 2015 | -0.281 | -0.4654 | No |
| 56 | RIF1 |  |  | 2016 | -0.281 | -0.4638 | No |
| 57 | RFC2 |  |  | 2044 | -0.284 | -0.4701 | No |
| 58 | YEATS4 |  |  | 2054 | -0.287 | -0.4711 | No |
| 59 | MAD2L2 |  |  | 2056 | -0.288 | -0.4697 | No |
| 60 | ZNF92 |  |  | 2057 | -0.288 | -0.4680 | No |
| 61 | TTF2 |  |  | 2066 | -0.290 | -0.4686 | No |
| 62 | GTSE1 |  |  | 2073 | -0.291 | -0.4687 | No |
| 63 | NUF2 |  |  | 2076 | -0.291 | -0.4676 | No |
| 64 | FOXM1 |  |  | 2081 | -0.291 | -0.4670 | No |
| 65 | TULP3 |  |  | 2105 | -0.298 | -0.4721 | No |
| 66 | CKAP2 |  |  | 2109 | -0.299 | -0.4712 | No |
| 67 | KIF11 |  |  | 2121 | -0.301 | -0.4727 | No |
| 68 | CTDSPL2 |  |  | 2132 | -0.303 | -0.4739 | No |
| 69 | SPAG9 |  |  | 2152 | -0.306 | -0.4777 | No |
| 70 | POLQ |  |  | 2169 | -0.308 | -0.4806 | No |
| 71 | ZGRF1 |  |  | 2170 | -0.308 | -0.4788 | No |
| 72 | RFC4 |  |  | 2185 | -0.312 | -0.4811 | No |
| 73 | DNA2 |  |  | 2205 | -0.314 | -0.4849 | No |
| 74 | TICRR |  |  | 2212 | -0.316 | -0.4848 | No |
| 75 | APBB2 |  |  | 2231 | -0.319 | -0.4883 | No |
| 76 | TIMELESS |  |  | 2236 | -0.320 | -0.4876 | No |
| 77 | DSN1 |  |  | 2255 | -0.325 | -0.4910 | No |
| 78 | CENPM |  |  | 2287 | -0.330 | -0.4983 | No |
| 79 | LIG1 |  |  | 2290 | -0.331 | -0.4969 | No |
| 80 | TIAM1 |  |  | 2303 | -0.334 | -0.4985 | No |
| 81 | NCAPG2 |  |  | 2335 | -0.340 | -0.5057 | No |
| 82 | ORC6 |  |  | 2343 | -0.342 | -0.5057 | No |
| 83 | CCNF |  |  | 2350 | -0.343 | -0.5055 | No |
| 84 | HJURP |  |  | 2360 | -0.346 | -0.5061 | No |
| 85 | POLA1 |  |  | 2363 | -0.347 | -0.5046 | No |
| 86 | STIL |  |  | 2376 | -0.349 | -0.5061 | No |
| 87 | FIGN |  |  | 2394 | -0.353 | -0.5091 | No |
| 88 | BLM |  |  | 2409 | -0.355 | -0.5112 | Yes |
| 89 | RNASEH2B |  |  | 2410 | -0.355 | -0.5091 | Yes |
| 90 | FANCI |  |  | 2411 | -0.356 | -0.5069 | Yes |
| 91 | PBK |  |  | 2412 | -0.356 | -0.5048 | Yes |
| 92 | SAP30 |  |  | 2413 | -0.356 | -0.5027 | Yes |
| 93 | MCM5 |  |  | 2424 | -0.359 | -0.5035 | Yes |
| 94 | CENPQ |  |  | 2430 | -0.361 | -0.5029 | Yes |
| 95 | RMI1 |  |  | 2432 | -0.362 | -0.5010 | Yes |
| 96 | ABAT |  |  | 2438 | -0.365 | -0.5003 | Yes |
| 97 | ARID1A |  |  | 2442 | -0.365 | -0.4991 | Yes |
| 98 | CENPJ |  |  | 2444 | -0.365 | -0.4972 | Yes |
| 99 | CEP192 |  |  | 2458 | -0.368 | -0.4989 | Yes |
| 100 | WDR76 |  |  | 2462 | -0.369 | -0.4976 | Yes |
| 101 | BRD8 |  |  | 2470 | -0.370 | -0.4974 | Yes |
| 102 | NCAPH |  |  | 2471 | -0.370 | -0.4952 | Yes |
| 103 | PSRC1 |  |  | 2485 | -0.375 | -0.4969 | Yes |
| 104 | PBRM1 |  |  | 2499 | -0.378 | -0.4985 | Yes |
| 105 | RSRC1 |  |  | 2501 | -0.379 | -0.4965 | Yes |
| 106 | LRP6 |  |  | 2505 | -0.379 | -0.4952 | Yes |
| 107 | PIP5K1B |  |  | 2506 | -0.379 | -0.4929 | Yes |
| 108 | BRD7 |  |  | 2513 | -0.381 | -0.4924 | Yes |
| 109 | EXO1 |  |  | 2516 | -0.381 | -0.4908 | Yes |
| 110 | SESTD1 |  |  | 2520 | -0.383 | -0.4894 | Yes |
| 111 | GLCCI1 |  |  | 2521 | -0.383 | -0.4871 | Yes |
| 112 | UHRF1 |  |  | 2549 | -0.388 | -0.4928 | Yes |
| 113 | TMEM106C |  |  | 2554 | -0.389 | -0.4917 | Yes |
| 114 | KNTC1 |  |  | 2558 | -0.389 | -0.4903 | Yes |
| 115 | CENPC |  |  | 2573 | -0.392 | -0.4921 | Yes |
| 116 | ATAD5 |  |  | 2574 | -0.392 | -0.4898 | Yes |
| 117 | BRIP1 |  |  | 2581 | -0.396 | -0.4892 | Yes |
| 118 | PBX3 |  |  | 2589 | -0.398 | -0.4890 | Yes |
| 119 | CBX5 |  |  | 2591 | -0.399 | -0.4869 | Yes |
| 120 | DST |  |  | 2614 | -0.404 | -0.4910 | Yes |
| 121 | SMC4 |  |  | 2634 | -0.408 | -0.4943 | Yes |
| 122 | ZWINT |  |  | 2641 | -0.410 | -0.4936 | Yes |
| 123 | STMN1 |  |  | 2646 | -0.411 | -0.4924 | Yes |
| 124 | PCM1 |  |  | 2654 | -0.413 | -0.4920 | Yes |
| 125 | DSEL |  |  | 2668 | -0.415 | -0.4934 | Yes |
| 126 | PDZRN3 |  |  | 2685 | -0.421 | -0.4957 | Yes |
| 127 | STAG2 |  |  | 2700 | -0.426 | -0.4973 | Yes |
| 128 | NPAT |  |  | 2708 | -0.428 | -0.4968 | Yes |
| 129 | ST8SIA4 |  |  | 2719 | -0.430 | -0.4973 | Yes |
| 130 | ELF2 |  |  | 2733 | -0.434 | -0.4985 | Yes |
| 131 | PRIM1 |  |  | 2739 | -0.436 | -0.4974 | Yes |
| 132 | CDK2 |  |  | 2746 | -0.439 | -0.4966 | Yes |
| 133 | HAT1 |  |  | 2750 | -0.441 | -0.4949 | Yes |
| 134 | RFC3 |  |  | 2765 | -0.446 | -0.4964 | Yes |
| 135 | MASTL |  |  | 2769 | -0.447 | -0.4946 | Yes |
| 136 | TOPBP1 |  |  | 2774 | -0.448 | -0.4932 | Yes |
| 137 | MSH2 |  |  | 2786 | -0.452 | -0.4938 | Yes |
| 138 | KIFC1 |  |  | 2788 | -0.452 | -0.4914 | Yes |
| 139 | FRYL |  |  | 2789 | -0.453 | -0.4887 | Yes |
| 140 | DNMT1 |  |  | 2794 | -0.455 | -0.4871 | Yes |
| 141 | RAD21 |  |  | 2797 | -0.457 | -0.4850 | Yes |
| 142 | DLEU2 |  |  | 2804 | -0.459 | -0.4841 | Yes |
| 143 | MCM7 |  |  | 2808 | -0.460 | -0.4822 | Yes |
| 144 | N4BP2 |  |  | 2813 | -0.461 | -0.4807 | Yes |
| 145 | CTCF |  |  | 2820 | -0.462 | -0.4797 | Yes |
| 146 | MCM6 |  |  | 2834 | -0.468 | -0.4808 | Yes |
| 147 | CENPF |  |  | 2842 | -0.472 | -0.4801 | Yes |
| 148 | FUS |  |  | 2845 | -0.474 | -0.4779 | Yes |
| 149 | KIF15 |  |  | 2855 | -0.478 | -0.4777 | Yes |
| 150 | ZNF680 |  |  | 2861 | -0.482 | -0.4763 | Yes |
| 151 | SMC2 |  |  | 2864 | -0.483 | -0.4740 | Yes |
| 152 | MMS22L |  |  | 2867 | -0.484 | -0.4717 | Yes |
| 153 | UBE2T |  |  | 2868 | -0.484 | -0.4689 | Yes |
| 154 | SPC25 |  |  | 2896 | -0.493 | -0.4740 | Yes |
| 155 | PIK3R3 |  |  | 2903 | -0.495 | -0.4728 | Yes |
| 156 | ILDR2 |  |  | 2904 | -0.496 | -0.4699 | Yes |
| 157 | BEST3 |  |  | 2911 | -0.498 | -0.4687 | Yes |
| 158 | BRCA2 |  |  | 2916 | -0.500 | -0.4669 | Yes |
| 159 | ATF7IP |  |  | 2920 | -0.502 | -0.4648 | Yes |
| 160 | MCM8 |  |  | 2935 | -0.507 | -0.4660 | Yes |
| 161 | NCAPG |  |  | 2951 | -0.514 | -0.4674 | Yes |
| 162 | CHD6 |  |  | 2952 | -0.514 | -0.4643 | Yes |
| 163 | ARX |  |  | 2960 | -0.520 | -0.4633 | Yes |
| 164 | E2F8 |  |  | 2961 | -0.520 | -0.4602 | Yes |
| 165 | MPHOSPH9 |  |  | 2964 | -0.523 | -0.4577 | Yes |
| 166 | IDH2 |  |  | 2966 | -0.524 | -0.4549 | Yes |
| 167 | WEE1 |  |  | 2974 | -0.527 | -0.4538 | Yes |
| 168 | NCAPD3 |  |  | 2986 | -0.532 | -0.4539 | Yes |
| 169 | SMARCA2 |  |  | 2988 | -0.533 | -0.4510 | Yes |
| 170 | MSI2 |  |  | 2992 | -0.537 | -0.4487 | Yes |
| 171 | SP3 |  |  | 2995 | -0.538 | -0.4461 | Yes |
| 172 | AGO2 |  |  | 2998 | -0.541 | -0.4435 | Yes |
| 173 | KLHL13 |  |  | 3000 | -0.542 | -0.4406 | Yes |
| 174 | PDLIM3 |  |  | 3011 | -0.546 | -0.4403 | Yes |
| 175 | TMPO |  |  | 3022 | -0.552 | -0.4400 | Yes |
| 176 | CHAF1A |  |  | 3023 | -0.552 | -0.4367 | Yes |
| 177 | ZNF43 |  |  | 3031 | -0.555 | -0.4355 | Yes |
| 178 | GMNN |  |  | 3046 | -0.561 | -0.4363 | Yes |
| 179 | NASP |  |  | 3049 | -0.563 | -0.4336 | Yes |
| 180 | TOP2B |  |  | 3059 | -0.567 | -0.4329 | Yes |
| 181 | RBL1 |  |  | 3063 | -0.569 | -0.4304 | Yes |
| 182 | MCM3 |  |  | 3064 | -0.570 | -0.4270 | Yes |
| 183 | SOX4 |  |  | 3066 | -0.571 | -0.4239 | Yes |
| 184 | SMC1A |  |  | 3067 | -0.571 | -0.4205 | Yes |
| 185 | LBR |  |  | 3068 | -0.572 | -0.4171 | Yes |
| 186 | RRM1 |  |  | 3070 | -0.574 | -0.4140 | Yes |
| 187 | RNF168 |  |  | 3075 | -0.576 | -0.4117 | Yes |
| 188 | ARL4D |  |  | 3076 | -0.577 | -0.4083 | Yes |
| 189 | CENPU |  |  | 3084 | -0.578 | -0.4069 | Yes |
| 190 | DEK |  |  | 3098 | -0.583 | -0.4073 | Yes |
| 191 | XRCC2 |  |  | 3104 | -0.588 | -0.4053 | Yes |
| 192 | CEP97 |  |  | 3109 | -0.592 | -0.4030 | Yes |
| 193 | CASP8AP2 |  |  | 3111 | -0.593 | -0.3998 | Yes |
| 194 | GINS2 |  |  | 3119 | -0.598 | -0.3983 | Yes |
| 195 | NRXN3 |  |  | 3127 | -0.603 | -0.3968 | Yes |
| 196 | MEIS1 |  |  | 3133 | -0.610 | -0.3946 | Yes |
| 197 | CENPK |  |  | 3134 | -0.610 | -0.3910 | Yes |
| 198 | GPC6 |  |  | 3138 | -0.612 | -0.3883 | Yes |
| 199 | HP1BP3 |  |  | 3139 | -0.612 | -0.3846 | Yes |
| 200 | MIS18BP1 |  |  | 3146 | -0.615 | -0.3827 | Yes |
| 201 | CDCA5 |  |  | 3167 | -0.636 | -0.3849 | Yes |
| 202 | PDS5B |  |  | 3177 | -0.641 | -0.3838 | Yes |
| 203 | BTG3 |  |  | 3184 | -0.647 | -0.3817 | Yes |
| 204 | RBBP8 |  |  | 3189 | -0.652 | -0.3790 | Yes |
| 205 | AASS |  |  | 3191 | -0.657 | -0.3754 | Yes |
| 206 | MYBL2 |  |  | 3196 | -0.660 | -0.3727 | Yes |
| 207 | NUSAP1 |  |  | 3199 | -0.663 | -0.3693 | Yes |
| 208 | CDK5RAP2 |  |  | 3205 | -0.672 | -0.3668 | Yes |
| 209 | DTL |  |  | 3210 | -0.679 | -0.3640 | Yes |
| 210 | PFN2 |  |  | 3215 | -0.685 | -0.3611 | Yes |
| 211 | CDCA7 |  |  | 3225 | -0.693 | -0.3596 | Yes |
| 212 | POLD3 |  |  | 3231 | -0.698 | -0.3570 | Yes |
| 213 | USP1 |  |  | 3233 | -0.699 | -0.3531 | Yes |
| 214 | CDK6 |  |  | 3234 | -0.704 | -0.3489 | Yes |
| 215 | LMNB1 |  |  | 3246 | -0.711 | -0.3480 | Yes |
| 216 | ASAP1 |  |  | 3247 | -0.711 | -0.3437 | Yes |
| 217 | RBBP4 |  |  | 3249 | -0.712 | -0.3398 | Yes |
| 218 | FEN1 |  |  | 3250 | -0.713 | -0.3355 | Yes |
| 219 | MCM4 |  |  | 3254 | -0.715 | -0.3322 | Yes |
| 220 | H1FX |  |  | 3261 | -0.721 | -0.3297 | Yes |
| 221 | MAGI1 |  |  | 3265 | -0.726 | -0.3262 | Yes |
| 222 | GAD1 |  |  | 3266 | -0.726 | -0.3219 | Yes |
| 223 | BCL11B |  |  | 3276 | -0.735 | -0.3202 | Yes |
| 224 | BARD1 |  |  | 3277 | -0.736 | -0.3158 | Yes |
| 225 | EZH2 |  |  | 3282 | -0.741 | -0.3126 | Yes |
| 226 | DLX6 |  |  | 3289 | -0.749 | -0.3099 | Yes |
| 227 | DHFR |  |  | 3303 | -0.765 | -0.3093 | Yes |
| 228 | CCNE2 |  |  | 3305 | -0.767 | -0.3050 | Yes |
| 229 | DUT |  |  | 3307 | -0.769 | -0.3007 | Yes |
| 230 | SOX2 |  |  | 3314 | -0.778 | -0.2979 | Yes |
| 231 | FBXW7 |  |  | 3315 | -0.779 | -0.2932 | Yes |
| 232 | RRM2 |  |  | 3317 | -0.783 | -0.2889 | Yes |
| 233 | GAS2L3 |  |  | 3321 | -0.790 | -0.2851 | Yes |
| 234 | PSIP1 |  |  | 3328 | -0.794 | -0.2821 | Yes |
| 235 | TYMS |  |  | 3333 | -0.798 | -0.2786 | Yes |
| 236 | BRCA1 |  |  | 3335 | -0.802 | -0.2741 | Yes |
| 237 | ARL6IP1 |  |  | 3339 | -0.804 | -0.2702 | Yes |
| 238 | H3F3A |  |  | 3342 | -0.807 | -0.2660 | Yes |
| 239 | TMSB15A |  |  | 3358 | -0.824 | -0.2655 | Yes |
| 240 | MCM10 |  |  | 3360 | -0.825 | -0.2609 | Yes |
| 241 | HELLS |  |  | 3361 | -0.826 | -0.2560 | Yes |
| 242 | RAD51AP1 |  |  | 3375 | -0.848 | -0.2548 | Yes |
| 243 | C21orf58 |  |  | 3383 | -0.861 | -0.2518 | Yes |
| 244 | ZEB2 |  |  | 3395 | -0.881 | -0.2498 | Yes |
| 245 | CDK1 |  |  | 3396 | -0.883 | -0.2446 | Yes |
| 246 | SP9 |  |  | 3411 | -0.914 | -0.2433 | Yes |
| 247 | FBXO5 |  |  | 3413 | -0.919 | -0.2381 | Yes |
| 248 | HMGB2 |  |  | 3414 | -0.922 | -0.2326 | Yes |
| 249 | PCNA |  |  | 3415 | -0.922 | -0.2271 | Yes |
| 250 | CCDC88A |  |  | 3421 | -0.944 | -0.2230 | Yes |
| 251 | PROM1 |  |  | 3436 | -0.974 | -0.2214 | Yes |
| 252 | ZNF704 |  |  | 3448 | -1.004 | -0.2187 | Yes |
| 253 | CHD7 |  |  | 3454 | -1.017 | -0.2141 | Yes |
| 254 | TOP2A |  |  | 3457 | -1.020 | -0.2086 | Yes |
| 255 | FAM111B |  |  | 3463 | -1.037 | -0.2040 | Yes |
| 256 | NEDD4L |  |  | 3469 | -1.045 | -0.1992 | Yes |
| 257 | ESCO2 |  |  | 3479 | -1.062 | -0.1956 | Yes |
| 258 | LCORL |  |  | 3490 | -1.089 | -0.1921 | Yes |
| 259 | RAB3IP |  |  | 3493 | -1.094 | -0.1862 | Yes |
| 260 | RGS12 |  |  | 3495 | -1.106 | -0.1799 | Yes |
| 261 | FAT1 |  |  | 3496 | -1.107 | -0.1733 | Yes |
| 262 | FAM111A |  |  | 3498 | -1.114 | -0.1669 | Yes |
| 263 | CLSPN |  |  | 3499 | -1.115 | -0.1603 | Yes |
| 264 | HIST1H1E |  |  | 3504 | -1.125 | -0.1548 | Yes |
| 265 | HIST1H1C |  |  | 3509 | -1.145 | -0.1492 | Yes |
| 266 | NCAM1 |  |  | 3514 | -1.168 | -0.1434 | Yes |
| 267 | SOX6 |  |  | 3517 | -1.174 | -0.1370 | Yes |
| 268 | SPECC1 |  |  | 3523 | -1.202 | -0.1313 | Yes |
| 269 | DCLK2 |  |  | 3524 | -1.206 | -0.1242 | Yes |
| 270 | ATAD2 |  |  | 3534 | -1.234 | -0.1195 | Yes |
| 271 | LSAMP |  |  | 3537 | -1.257 | -0.1126 | Yes |
| 272 | FBLN1 |  |  | 3539 | -1.262 | -0.1054 | Yes |
| 273 | CDH2 |  |  | 3543 | -1.297 | -0.0986 | Yes |
| 274 | CDCA7L |  |  | 3557 | -1.393 | -0.0941 | Yes |
| 275 | ZBTB20 |  |  | 3567 | -1.435 | -0.0883 | Yes |
| 276 | SYNE2 |  |  | 3574 | -1.510 | -0.0811 | Yes |
| 277 | HIST1H1B |  |  | 3592 | -1.679 | -0.0761 | Yes |
| 278 | DLX1 |  |  | 3593 | -1.696 | -0.0660 | Yes |
| 279 | DLX2 |  |  | 3595 | -1.720 | -0.0561 | Yes |
| 280 | HIST1H1D |  |  | 3596 | -1.757 | -0.0456 | Yes |
| 281 | ASCL1 |  |  | 3604 | -1.822 | -0.0369 | Yes |
| 282 | INSM1 |  |  | 3607 | -1.872 | -0.0263 | Yes |
| 283 | DLX5 |  |  | 3623 | -2.501 | -0.0159 | Yes |
| 284 | LMO1 |  |  | 3635 | -3.371 | 0.0009 | Yes |
Table: GSEA details [plain text format]

  

Fig 2: RICHARDS\_NOWAKOWSKI\_SCIENCE\_2017\_MGE\_IPC2\_UPREG: Random ES distribution      
 Gene set null distribution of ES for **RICHARDS\_NOWAKOWSKI\_SCIENCE\_2017\_MGE\_IPC2\_UPREG**
